# Prevalence and clinical profile of comorbidity among newly diagnosed pulmonary tuberculosis patients: a multi-center observational study in eastern China

**DOI:** 10.3389/fmed.2025.1446835

**Published:** 2025-01-28

**Authors:** Wei Wang, Xiaomeng Wang, Songhua Chen, Jun Li, Qinglin Cheng, Yu Zhang, Qian Wu, Kui Liu, Xuli Jiang, Bin Chen

**Affiliations:** ^1^Department of Tuberculosis Control and Prevention, Zhejiang Provincial Center for Disease Control and Prevention, Hangzhou, Zhejiang, China; ^2^Zhejiang Key Lab of Vaccine, Infectious Disease Prevention and Control, Hangzhou, Zhejiang, China; ^3^Zhejiang Provincial Key Laboratory for Vaccine and Infectious Disease Prevention and Control, Hangzhou, Zhejiang, China; ^4^Hangzhou Center for Disease Control and Prevention, Hangzhou, Zhejiang, China; ^5^The Quzhou Affiliated Hospital of Wenzhou Medical University (Quzhou People’s Hospital), Quzhou, Zhejiang, China

**Keywords:** pulmonary tuberculosis, comorbidity, prevalence, tuberculosis, observational study

## Abstract

**Objective:**

To identify the composition of comorbidities among patients with newly diagnosed pulmonary tuberculosis and assess the impact of comorbidities on the clinical characteristics of patients.

**Methods:**

This study was conducted in 13 hospitals across 13 counties in Zhejiang province, China. Patient data collected in this study included demographic characteristics, chest radiography results, etiological results, and comorbidities. Descriptive statistics were conducted to describe the composition of comorbidities of all participants. Univariate and multivariate logistic regression analyzes were performed to identify the effects of comorbidities on the clinical features of the participants.

**Results:**

Of the 8,421 total participants, 27.6% reported cavities in the chest radiography results, 41.9% were *Mycobacterium tuberculosis*-positive in the etiology test results, and 38.7% (3,258/8,421) had at least one type of comorbidity. The most predominant comorbidity was pleuritis (1,833, 21.8%), followed by diabetes mellitus (763, 9.1%), other extrapulmonary tuberculosis (421, 5%), tracheobronchial tuberculosis (275, 3.3%), and silicosis (160, 1.9%). Participants with diabetes mellitus had the highest rate of chest cavities on X-ray (54.8%), followed by those with silicosis (33.1%). In addition, a higher percentage of the *M. tuberculosis*-positive etiology (45%) was observed in participants without comorbidities than in participants with comorbidities (37.1%). Compared to patients without comorbidities, patients with diabetes mellitus (adjusted odds ratio [AOR]: 2.88, 95% confidence interval [CI]: 2.42–3.43) were more likely to show cavities in chest X-ray, while patients with pleuritis (AOR: 0.27, 95% CI: 0.23–0.32), other extrapulmonary tuberculosis (AOR: 0.48, 95% CI: 0.36–0.64), and tracheobronchial tuberculosis (AOR: 0.40–0.79) were less likely to show chest cavities in X-ray. In addition, patients with diabetes mellitus (AOR: 2.05, 95% CI: 1.72–2.45), tracheobronchial tuberculosis (AOR: 3.22, 95% CI: 2.4–4.32) were more likely to show *Mycobacterium tuberculosis*-positive in the etiology, and patients with pleuritis (AOR: 0.25, 95% CI: 0.22–0.29), other extrapulmonary tuberculosis (AOR: 0.61, 95% CI: 0.48–0.76) were less likely to show *Mycobacterium tuberculosis*-positive in the etiology.

**Conclusion:**

The prevalence of comorbidities was high in patients newly diagnosed with pulmonary tuberculosis. Thus, integration of screening and personalized management is needed for the control of tuberculosis and its comorbidities.

## 1 Introduction

In 2022, an estimated total of 10.6 million people worldwide were ill with tuberculosis (TB), with 1.13 million TB-associated deaths (1, 2). It has been estimated that a quarter of the world’s population has been infected with TB (3), and approximately 5–15% of these infections will progress to active TB during the patient’s lifetime (2). TB is a serious public health threat. To effectively address this problem through a multisectoral response, the End TB Strategy set the target of a 90% reduction in TB incidence and a 95% reduction in TB mortality between 2015 and 2035 (1, 4), as proposed by the World Health Organization (WHO) and approved by the 67th World Health Assembly.

Over the past several decades, tremendous progress has been achieved in TB control after the implementation of a series of measures based on different TB control strategies, including directly observed treatment, short-course (DOTS) strategies, the Stop TB Strategy, and the Global Plan to Stop TB (4, 5). In the past, TB services were limited due to inadequate healthcare capacity and low economic levels in geographic regions of focus, and these efforts were typically focused on single conditions while neglecting to identify and manage other coexisting conditions (6). TB is a chronic inflammatory disease that may increase a person’s susceptibility to other non-communicable and communicable diseases, such as diabetes mellitus (DM), depression, malaria, and human immunodeficiency virus (HIV)/acquired immunodeficiency syndrome (AIDS) (7, 8). Other conditions that commonly co-occur with TB may worsen its clinical course, affect the efficacy of TB treatment, and increase the risk of relapse.

The proportion of people with coexisting medical problems is increasing as the global population is aging (9), which not only increases the challenges of practicing clinicians but also places significant health and financial burdens on patients with TB and health services (10, 11). Accordingly, TB comorbidities have received increasing attention in recent years (6). An exploratory survey of 27 high-TB burden countries identified HIV, DM, depression, and tobacco and alcohol use disorders as the most common comorbid conditions in TB (12). Several studies have been conducted to estimate the prevalence of different comorbidities in patients with TB. The global prevalence of DM in TB patients is approximately 15% (13). The prevalence of TB and noncommunicable disease comorbidity was 26.9% among public primary care patients in South Africa (14). A World Health Survey conducted in 48 low- and middle-income countries showed that the prevalence of one or more comorbid noncommunicable diseases was 68.8% in individuals with TB (8). The coexistence of silicosis and TB was also observed in many studies, where the incidence of TB in patients with silicosis is higher than the incidence of TB in the general population (15, 16).

Although there have been some research reports on TB comorbidity in China (11, 17, 18), most of these studies focused on TB and DM comorbidity only (19–22), and further evidence is needed to guide the optimization of TB prevention and control policies. The objective of this study was to identify and describe the composition of comorbidities among newly diagnosed patients with TB, and to assess the impact of comorbidities on the clinical characteristics of TB in real-world settings as based on patients from 13 designated TB hospitals across Zhejiang Province, China.

## 2 Methods

### 2.1 Study design

The study was conducted from January 1, 2017 to February 28, 2019 in 13 hospitals across 13 counties in Zhejiang province, eastern China. Patients with pulmonary TB (PTB) were newly diagnosed as outpatients, and drug-susceptible and HIV-negative patients were included in the study. Patients with retreated, drug-resistant, or HIV-positive TB were excluded. With informed consent from all eligible primary TB patients, investigations were performed by trained medical personnel.

The data collected in this study included basic demographic characteristics (gender, age, ethnicity, occupation, household registration), chest radiography examination results, and etiological examination results of the participants. In addition, we collected information on whether the participants had any of the following diseases: Pleuritis, tracheal bronchial TB, DM, silicosis, or other extrapulmonary TB, which clinicians believe may affect the course or effectiveness of TB treatment.

### 2.2 Study definitions

Patients with confirmed PTB based on the National Diagnostic Criteria for TB (WS 288–2017) and the Classification of TB criteria (WS196–2017) were included in the study. Laboratory diagnosis was based on the results of detectable acid-fast bacilli obtained from sputum smears and/or sputum cultures, or a positive result from molecular diagnosis. The clinical diagnosis was based on chest radiographs, epidemiological surveys, clinical symptoms, and other relevant tests. Since pleurisy and tracheobronchial TB typically occur outside the lungs and the treatment regimen is different from that of PTB, these two diseases were classified as concurrent diseases of PTB in this study. The comorbidities investigated in this study included pleuritis, tracheobronchial TB, DM, silicosis, and other extrapulmonary TB, which were diagnosed by physicians based on national diagnostic criteria, or as reported by participants whether they had been diagnosed with one or more diseases in another healthcare facility prior to the present TB visit.

### 2.3 Statistical analysis

Descriptive statistics were used to demonstrate participant characteristics. UpSet plots were generated using the R package UpSetR to describe the comorbidities of all participants (23). Chi-square test was used to explore the correlation between comorbidity and demographic characteristics. The results of chest radiography and etiology were used to represent the clinical characteristics of PTB in this study. If cavity was reported in chest X-ray examination results or positive was reported in *Mycobacterium tuberculosis* in etiology test, which were usually considered to be serious clinical manifestations of TB that require the focus of clinicians. In this study, the cavity in chest X-ray examination results, positive in *Mycobacterium tuberculosis* etiology test were taken as dependent variables, and comorbidities were taken as independent variables to identify the effects of comorbidities on the participants’ clinical features by multivariate logistic regression analyses. In the multivariate logistic regression analysis, the effects of sex, age, ethnicity, occupation, and household registration were adjusted. The estimation of their odds ratios (ORs) and 95% confidence intervals (CIs) were determined. Statistical significance was set at *p* < 0.05.

## 3 Results

### 3.1 Characteristics of newly diagnosed PTB patients

A total of 8,421 participants participated in the study, the characteristics of which are presented in Table 1. Of this total, 68.1% were female and 67.0% were from local provincial residences. Farmers accounted for the largest proportion (39.1%) of participants, followed by workers (18.9%), the unemployed (11.1%), migrant workers (9.3%), and business/service workers (5.8%). The predominant age group was ages 25–35 years (21.4%), followed by age 15–25 years (18.4%), age 45–55 years (15.0%), and age 35–45 years (13.9%). Ethnic Han participants were the most common ethnicity (97.9%). Of all the participants, 27.6% showed cavities in the chest X-ray examination results, and 41.9% were *Mycobacterium tuberculosis*-positive in the etiology test results.

**Table 1:** The characteristics of newly pulmonary tuberculosis patients.

Items	Numbers	Proportion (%)
Gender		
Male	5,736	68.10
Female	2,685	31.90
Age group (years)		
≤15	59	0.70
16–25	1,551	18.40
26–35	1,805	21.40
36–45	1,168	13.90
46–55	1,267	15.00
56–65	1,031	12.20
66–75	875	10.40
≥76	665	7.90
Ethnicity		
Others	177	2.10
Han	8,244	97.90
Occupation		
Unemployed	933	11.10
Students	346	4.10
Public institutions or civil servants	283	3.40
Business/service workers	487	5.80
Workers	1,591	18.90
Migrant workers	783	9.30
Farmers	3,295	39.10
Retirees	346	4.10
Others	357	4.20
Registered residence		
Local province	5,642	67.00
Other provinces	2,779	33.00
Chest X-ray examination results		
No cavity	6,100	72.4
cavity	2,321	27.6
The results of *Mycobacterium tuberculosis* positive in etiology test		
non-positive	4,889	58.1
Positive	3,532	41.9
Whether participants had comorbidities		
No	5,163	61.30
Yes	3,258	38.70

### 3.2 Prevalence of comorbidities among newly diagnosed PTB patients

Of all the newly diagnosed PTB patients included in our study, 38.7% (3,258/8,421) had at least one comorbidity. Approximately 40% of male participants and 35.9% of female participants reported having investigated comorbidities. The proportion of patients with comorbidities in the overall population of PTB patients increased with age. The highest proportion of comorbidities was observed in participants aged 75 years and older (54.6%), followed by the 65–75 years age group (53.2%), 55–65 years age group (49.8%), 45–55 years age group (45.8%), 35–45 years age group (33.6%), and 15–25 years age group (28.8%). The proportion of comorbidities in local provincial residences was 43.2 and 29.6% in participants with household registration outside the province. The proportions of comorbidities in retirees, farmers, workers, business and service workers were 56.1, 46.8, 28.4, 25.7, and 27.2%, respectively.

**Figure 1:**
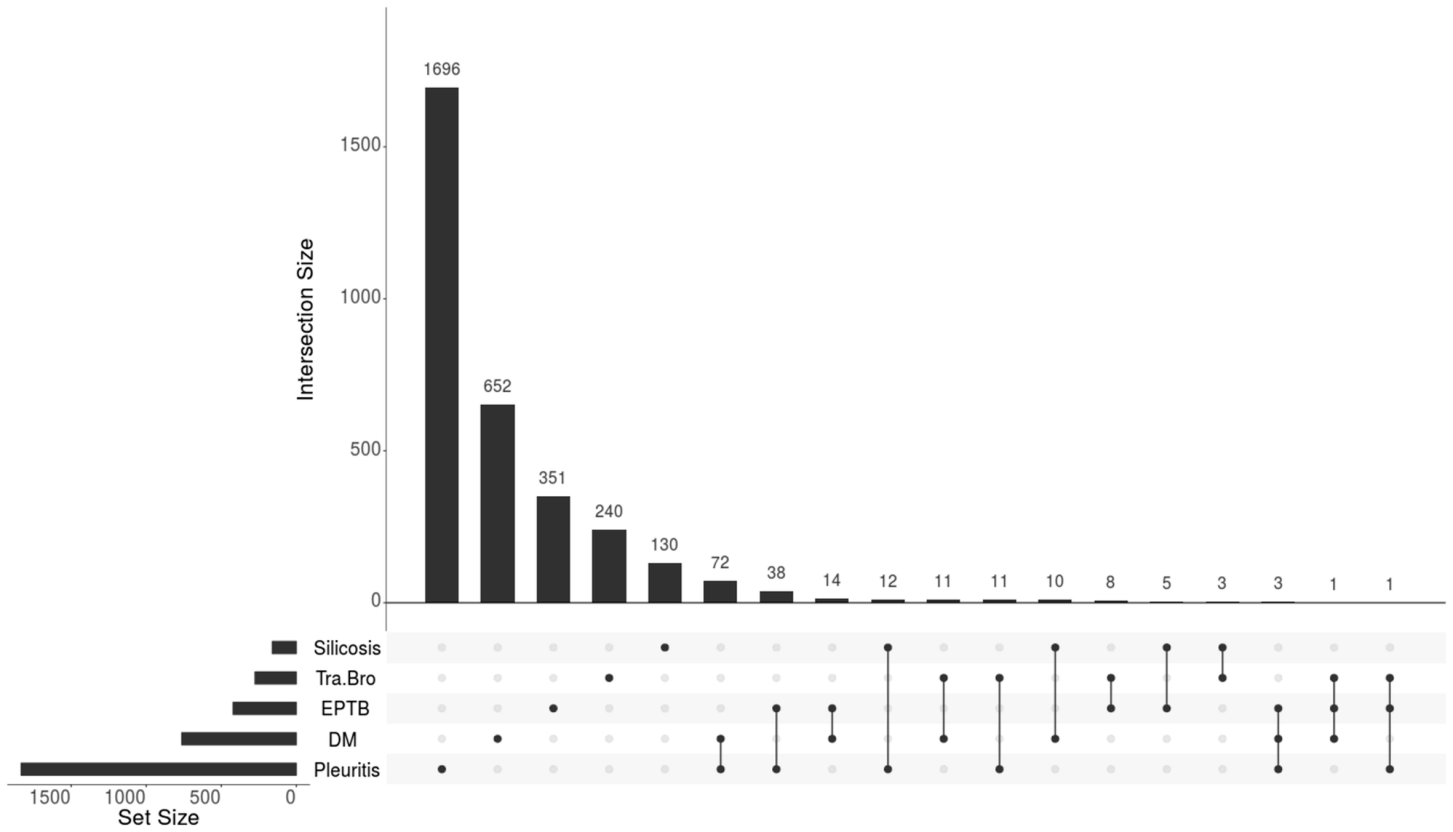
The composition of comorbidities in newly pulmonary tuberculosis patients (*N* = 3,258). Tra-Bro, Tracheobronchial tuberculosis; EPTB, Other Extrapulmonary Tuberculosis; DM, Diabetes Mellitus.

The predominant type of comorbidity was pleuritis (1,833, 21.8%), followed by DM (763, 9.1%), other extrapulmonary TB (421, 5%), tracheobronchial TB (275, 3.3%), and silicosis (160, 1.9%), which are shown in Figure 1. Among the 3,069 participants with only one type of comorbidity, 1,696 had pleuritis, 652 had DM, 351 had other extrapulmonary TB, 240 had tracheobronchial TB, and 130 had silicosis. Among the 189 participants with two or more comorbidities, 72 had both pleuritis and DM, 38 had both pleuritis and other extrapulmonary TB, 14 had both DM and other extrapulmonary TB, and 5 had three or more comorbidities.

### 3.3 Clinical profiles of comorbidity among newly diagnosed PTB patients

The clinical profiles of comorbidities in patients with newly diagnosed PTB are shown in Table 2. Of the 5,163 newly diagnosed TB patients without comorbidities in this study, 30.6% showed cavities in the chest X-ray examination results, which was higher than that of participants in the comorbidities group (22.7%). Participants with DM had the highest rate of chest cavities on X-ray (54.8%), followed by those with silicosis only (33.1%). In addition, a higher percentage of *M. tuberculosis*-positive etiology (45%) was observed in participants without comorbidities than in participants with comorbidities (37.1%). Among the 3,069 participants who had only one type of comorbidity, the proportion of *M. tuberculosis*-positive etiology was highest in participants with tracheobronchial TB (72.5%), followed by participants with DM (67.8%).

**Table 2:** The correlation between comorbidity and demographic characteristics of newly pulmonary tuberculosis patients.

Items	Co-morbidities		*χ^2^*	*p*
	No (%)	Yes (%)		
Gender			12.90	<0.001
Male	3,442 (60.0)	2,294 (40.0)		
Female	1,721 (64.1)	964 (35.9)		
Age group (years)			398.33	<0.001
≤15	43 (72.9)	16 (27.1)		
16–25	1,104 (71.2)	447 (28.8)		
26–35	1,315 (72.9)	490 (27.1)		
36–45	775 (66.4)	393 (33.6)		
46–55	687 (54.2)	580 (45.8)		
56–65	518 (50.2)	513 (49.8)		
66–75	419 (47.9)	456 (52.1)		
≥76	302 (45.4)	363 (54.6)		
Ethnicity			12.29	<0.001
Others	131 (74)	46 (26.0)		
Han	5,032 (61)	3,212 (39.0)		
Occupation			269.73	<0.001
Unemployed	590 (63.2)	343 (36.8)		
Students	252 (72.8)	94 (27.2)		
Public institutions or civil servants	185 (65.4)	98 (34.6)		
Business/service workers	362 (74.3)	125 (25.7)		
Workers	1,139 (71.6)	452 (28.4)		
Migrant workers	511 (65.3)	272 (34.7)		
Farmers	1,752 (53.2)	1,543 (46.8)		
Retirees	152 (43.9)	194 (56.1)		
Others	220 (61.6)	137 (38.4)		
Registered residence			145.12	<0.001
Local province	3,206 (56.8)	2,436 (43.2)		
Other provinces	1,957 (70.4)	822 (29.6)		
Chest X-ray results				
No cavity	3,582 (58.7)	2,518 (41.3)	62.57	<0.001
Cavity	1,581 (68.1)	740 (31.9)		
*mycobacterium tuberculosis* positive test				
non-positive	2,840 (58.1)	2,049 (41.9)	51.00	<0.001
Positive	2,323 (65.8)	1,209 (34.2)		

Non-positive: negative or no etiological results.

### 3.4 Factors associated with comorbidities of newly diagnosed PTB patients

The results of chi-square analysis showed that significant statistical difference of comorbidities was observed in different gender, age, ethnicity, occupation, household registration (Table 2), respectively. In multivariate logistic analysis, when compared to patients without comorbidities (Table 3), patients with DM (AOR: 2.88, 95% CI: 2.42–3.43) were at a higher risk for observed cavities in chest X-ray, while patients with pleuritis (AOR: 0.27, 95% CI: 0.23–0.32), other extrapulmonary TB (AOR: 0.48, 95% CI: 0.36–0.64), and tracheobronchial TB (AOR:0.56, 95% CI: 0.40–0.79) were at a lower risk for observed cavities in chest X-ray. In addition, with respect to the proportion of patients with *M. tuberculosis*-positive etiology, a higher risk was observed in patients with DM (AOR: 2.05, 95% CI: 1.72–2.45) and tracheobronchial TB (AOR: 3.22, 95%: 2.40–4.32), and a lower risk in patients with pleuritis (AOR: 0.25, 95% CI: 0.22–0.29) and extrapulmonary TB (AOR: 0.61, 95%: 0.48–0.76).

**Table 3:** The analysis of the influence of different co-morbidities on the clinical features of newly pulmonary tuberculosis.

Items		Cavity was reported in chest X-ray examination results				Positive of *Mycobacterium tuberculosis* in etiology test			
		Count	Percent (%)	OR (95% CI)	AOR (95% CI)	Count	Percent (%)	OR (95% CI)	AOR (95% CI)
What kinds of comorbidities did the participants have?									
No comorbidity	5,163	1,581	30.60	1	1	2,323	45.0	1	1
Pleuritis	1,696	190	11.20	0.29 (0.24–0.34)	0.27 (0.23–0.32)	325	19.2	0.29 (0.25–0.33)	0.25 (0.22–0.29)
Diabetes mellitus	652	357	54.80	2.74 (2.32–3.23)	2.88 (2.42–3.43)	442	67.8	2.57 (2.16–3.06)	2.05 (1.72–2.45)
Silicosis	130	43	33.10	1.12 (0.77–1.62)	0.91 (0.63–1.33)	58	44.6	0.99 (0.69–1.40)	0.73 (0.51–1.04)
Other extrapulmonary tuberculosis	351	60	17.10	0.47 (0.35–0.62)	0.48 (0.36–0.64)	121	34.5	0.64 (0.51–0.81)	0.61 (0.48–0.76)
Tracheobronchial tuberculosis	240	41	17.10	0.47 (0.33–0.66)	0.56 (0.40–0.79)	174	72.5	3.22 (2.42–4.30)	3.22 (2.40–4.32)
At least two of the five comorbidities	189	49	25.90	0.79 (0.57–1.10)	0.81 (0.58–1.14)	89	47.1	1.09 (0.81–1.46)	0.87 (0.64–1.17)

OR, odds ratio in univariate logistic regression analysis; CI, confidence interval; AOR, adjusted odds ratio in multivariate logistic regression analysis, adjusted for the effects of gender, age, ethnicity, occupation, and household registration.

## 4 Discussion

This study investigated the prevalence of five comorbidities that may affect the treatment course of PTB. We found that the prevalence of the five comorbidities investigated was as high as 38.7% in patients with newly diagnosed PTB. Pleurisy and DM were the most common comorbidities. Compared to PTB patients without any comorbidities, the risk of pulmonary cavity in X-ray and positive in *Mycobacterium tuberculosis* etiology test was higher in PTB patients complicated with DM and lower in those complicated with pleurisy or other extrapulmonary TB.

PTB comorbidities are commonly reported in low- and middle-income countries, and can include non-communicable diseases (NCD), chronic communicable diseases, and mental disorders (24). These comorbid conditions pose a great challenge for both individual health and healthcare systems. In this study, comorbidities were observed in nearly 40% of all participants. Another study in China reported common comorbidities such as DM and hypertension among patients with PTB (17). In India, more than half of PTB patients reported multimorbidity, with depression, DM, acid peptic disease, and hypertension being the most common comorbidities (25). Among the five investigated comorbidities in our study, the predominant form of comorbidity was pleuritis, followed by DM, extrapulmonary TB, tracheobronchial TB, and silicosis. Additionally, pleuritis, and DM, pleuritis and other extrapulmonary TB, DM and other extrapulmonary TB were the most commonly clustering comorbidities. Multimorbidity was also observed in our study. The clustering of different comorbidities greatly increases the complexity of treatment and management and adversely affects health, economic, and mortality outcomes (6), threatening the capacity to intervene in a global TB epidemic. Evidence-based frameworks for integration and person-centered care, screening approaches, and effective interventions have been demonstrated to help control for TB comorbidities (26). Thus, integrating screening and management of these comorbidities within TB programs and addressing TB multimorbidity in policy and practice are essential to meet the End TB targets (12).

This study demonstrated that the proportion of patients with PTB comorbidities increased with age and was higher in male patients than in female patients. A study conducted in South Africa on NCD multimorbidity among TB patients in public primary care clinics also reported that the likelihood of multimorbidity was higher among older patients (14). In India and 48 other low- and middle-income countries, increasing age was also significantly associated with multimorbidity among patients with TB (8, 25). Studies have shown that older people are more susceptible to infections and other chronic diseases (27), which may be related to a decline in the immune system (28). A study in South Korea revealed an increasing prevalence of comorbidities among female TB patients with increasing age (29). Differences in diseases between male and female patients may be influenced by differences in sex hormones, sex-related genetic backgrounds, genetic regulation and metabolism, and differing living habits (30). This study also found that retirees and farmers have relatively high comorbidity rates. Generally, retirees are older, so similar results as those of older TB patients are understandable. Farmers are more likely to face socioeconomic deprivation (31), such as a lack of education, low income, overcrowding, and poor nutrition, which may increase the risk of disease. These findings acknowledge the need for the personalized management of TB, and evaluation of the risks for comorbidities should consider the sex, age, and occupation of the patient (6, 29).

TB pleurisy is a common form of extrapulmonary TB (32, 33) with exceptionally low sensitivity to *M. tuberculosis*. This study found that the risks of pulmonary cavity and etiological positivity were lower in participants with TB pleurisy or other types of extrapulmonary TB. A previous study showed that TB pleural effusion is mostly a hypersensitivity reaction (34) and is rarely positive for acid-fast bacilli staining. In addition, obtaining high-quality sputum samples from patients with TB pleurisy is difficult. Similar to the findings of this study, most studies have reported no specific lung or pleural radiological findings that can confirm TB pleurisy (34–36). Multiple factors make the timely diagnosis of TB pleurisy challenging. Therefore, more in-depth scientific research is required for the early detection of extrapulmonary TB, such as TB pleurisy.

DM has been considered one of the two most common NCDs to coexist with TB (6, 13). The prevalence of DM among TB patients in various low- and middle-income countries varies from 1.8 to 45% (37). In a systematic review and meta-analysis of the compiled data from 2.3 million people suffering from active TB, the global prevalence of DM among patients with TB was estimated to be 15% (13), which was slightly higher than the 9% found in this study. Heterogeneity may be the result of disparities in age, gender, region, level of income, and development between the study regions. The results of this study showed that the risk of pulmonary cavity disease and etiological positivity were higher in patients with pulmonary TB complicated by DM, which is consistent with previous studies in China (21, 22), Saudi Arabia (38), and Qatar (39). DM can lead to a greater risk of poor TB outcomes by weakening the immune system, resulting in worse clinical presentation, more symptoms, and death (37, 40). Similarly, better DM control in patients with TB could improve TB treatment success and reduce TB transmission, averting millions of TB cases and deaths (41). Accordingly, in 2011, the WHO declared a framework for the care and control of TB and DM (42), which recommends the collaborative care of TB-related DM.

This study has some limitations. First, only five diseases were investigated in this study at the time of the initial patient visit, which may have introduced differences in the number and type of comorbidities compared to those of other studies. Second, some of the participants’ comorbidities were self-reported based on their historical diagnosis, which may have been biased by recall. Thirdly, the results of this study may be partially overestimated due to the limitations of cross-sectional studies. Despite these limitations, this was a multicenter observational study covering 13 designated hospitals from 13 counties across Zhejiang province, China, with results that could reflect comorbidities among newly diagnosed PTB patients in eastern coastal China and provide valuable scientific insight to policy makers to optimize the prevention and control of TB comorbidities in the future.

## 5 Conclusion

The prevalence of the investigated comorbidities was high in patients with newly diagnosed PTB. Among the five comorbidities investigated, pleurisy and DM were the most common comorbidity. The risk of pulmonary cavity and etiological positivity was higher in patients with PTB and DM. Thus, an integration of screening and personalized management is needed in the control of TB and its comorbidities.

## Data Availability

The raw data supporting the conclusions of this article will be made available by the authors, without undue reservation.
